# 3D-Printed Colloidal Crystal Hydrogel Crown Fused with Machine Learning-Integrated Resistance Strain Sensor for Pressure Sensing

**DOI:** 10.34133/bmr.0313

**Published:** 2026-02-04

**Authors:** Zheng Mao, Dongxiang Yang, Ling Tang, Qing He, Yue Wang, Songchao Fu, Zhiwei Jiang, Ying Wang, Chenkai Zou, Cihui Liu, Linling Yin

**Affiliations:** ^1^ Center for Future Optoelectronic Functional Materials, School of Computer and Electronic Information/School of Artificial Intelligence, Nanjing Normal University, Nanjing 210046, China.; ^2^ Department of Nursing, Shanghai General Hospital, Shanghai Jiao Tong University School of Medicine, Shanghai 201600, China.; ^3^ Department of Stomatology, Shanghai General Hospital, Shanghai Jiao Tong University School of Medicine, Shanghai 200080, China.

## Abstract

Advancements in dental restoration technologies have created transformative opportunities for enhancing crown repair through intelligent sensing and adaptive design. While modern materials like ceramics and resins improve aesthetic and functional outcomes, persistent challenges in long-term fit, durability, and dynamic pressure monitoring remain unaddressed. This study introduces a groundbreaking approach that synergizes 3-dimensional (3D)-printed colloidal crystal hydrogel crowns with machine learning-integrated resistance strain sensors. The hydrogel’s inverse opal structure ensures robust adhesion to the tooth surface, while embedded strain sensors capture real-time, multidirectional pressure data. Unlike conventional sensing systems, our framework employs machine learning algorithms to dynamically interpret strain patterns, enabling predictive modeling of occlusal forces and adaptive calibration of crown fit. The hydrogel’s temperature-responsive properties, combined with sensor stability under oral environmental fluctuations, ensure reliable long-term performance. Machine learning further enhances diagnostic precision by correlating strain-resistance data with clinical parameters, facilitating personalized adjustments to restoration plans. This work pioneers the fusion of intelligent sensing, material innovation, and data-driven analytics in dental care, establishing a foundation for next-generation adaptive and patient-specific restorative solutions.

## Introduction

Advancements in dental restoration technologies have created new opportunities and challenges in the field of tooth crown repair [1–4]. Over the past few decades, crown design and fabrication have evolved from traditional metal materials to more advanced options such as ceramics, resins, and composite materials [5–10]. These modern materials have greatly improved the aesthetic and functional qualities of crowns. However, issues related to long-term fit, durability, and comfort still persist. Recently, the rapid development of 3-dimensional (3D) printing and smart sensor technologies has opened new possibilities for personalized crown fabrication, providing unprecedented opportunities to enhance dental restoration techniques [11–16]. Particularly, the application of smart sensors for precise monitoring of stress distribution and fit during the crown restoration process offers promising directions for clinical practice [17–19].

While 3D printing technology has made notable progress in the dental field, most current research in crown restoration focuses primarily on optimizing materials and design [20]. Many studies have explored the development of high-strength, aesthetically pleasing dental materials, such as all-ceramic crowns and 3D-printed resin crowns, which can be custom-made using precise 3D printing techniques. Despite marked advancements in the aesthetics and functionality of these crown materials, there remains a lack of accurate monitoring of the stress and mechanical fit between the crown and the tooth during restoration [21]. This limitation can lead to discomfort or restoration failure for patients in the long term.

To address this gap, some researchers have begun exploring the integration of sensor technologies in crown restorations. In recent years, sensor-based research in dentistry has become an emerging area of interest [22,23]. For example, several studies have embedded force sensors within crowns to monitor pressure changes during chewing. These studies often use piezoelectric or strain sensors to detect pressure variations between the crown and the tooth. Zhu and colleagues [5] developed a high-precision biomimetic crown using an extrusion-based 3D printing technique, employing a shear-induced approach to construct hierarchical, highly ordered structures. However, these studies primarily focus on the additive manufacturing of crowns and fail to address the compatibility issues between the materials and sensors, nor do they explore how to improve the stability and biocompatibility of sensors in real-world applications [24–26].

Similarly, other studies have worked on developing embedded sensor systems to monitor the fit between crowns and teeth. For instance, Yang and colleagues [27] introduced a compact, wireless, passive sensor system capable of continuously monitoring orthodontic forces. Unlike traditional large, wired orthodontic force measurement devices, this highly integrated flexible sensor system can be placed entirely within the oral cavity for long-term, real-time monitoring. However, these studies often lack a comprehensive approach to integrating sensor technology with the materials of the crown itself, particularly in optimizing sensor design and installation for different tooth shapes and sizes.

**Fig. 1.**
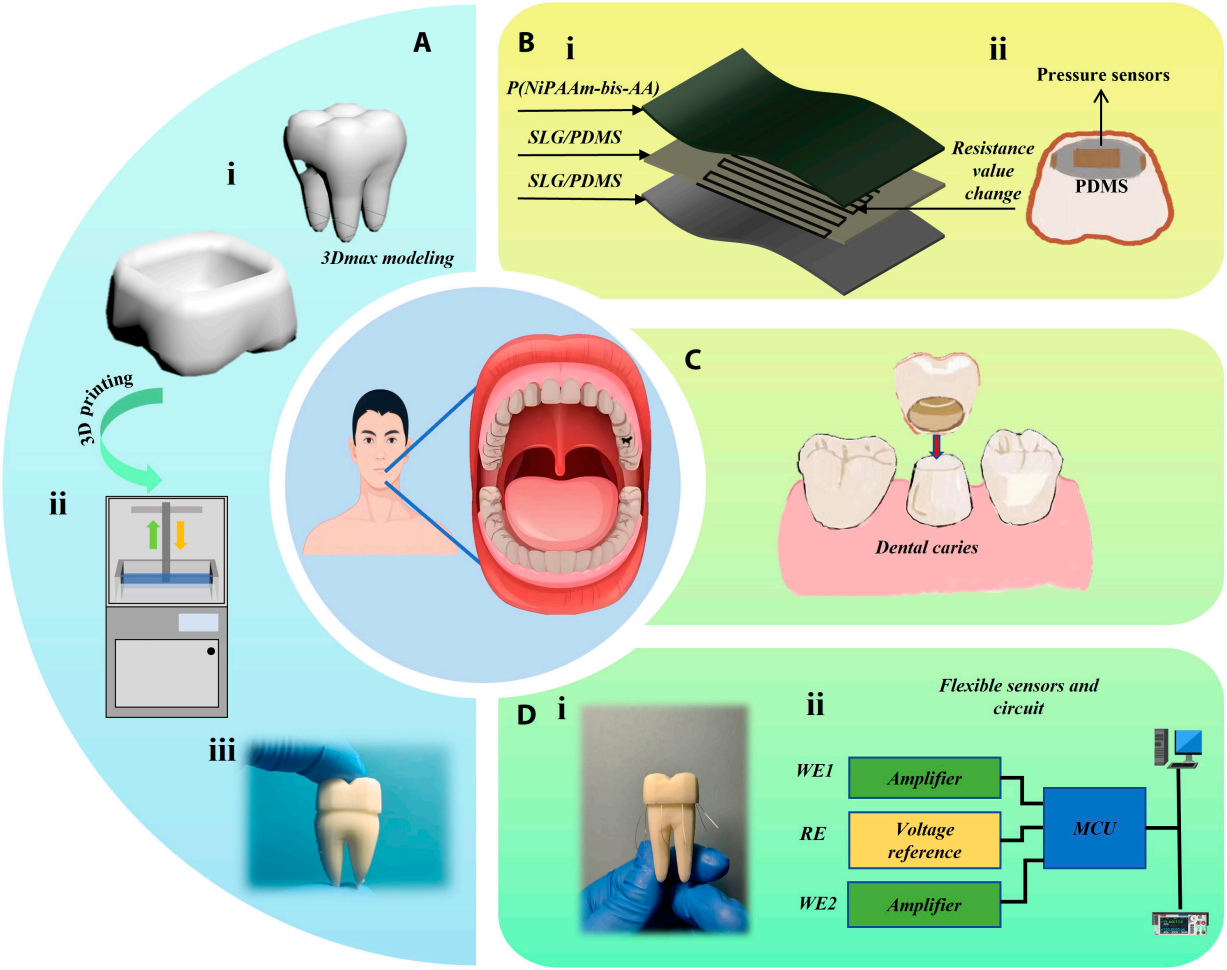
Dental restoration and health monitoring system. (A) Process diagram for printing dental crown models using 3D printing technology. (i) Schematic diagram of the model after 3Dmax modeling. (ii) Schematic diagram of 3D printer. (iii) Physical image of resin model. (B) (i) Schematic diagram of the structure of a flexible pressure resistance sensor. (ii) Schematic diagram of a dental crown model embedded with a flexible pressure resistance sensor. (C) Schematic diagram of the process of repairing dental caries using a dental crown model embedded with flexible pressure sensors. (D) (i) Physical image of dental crown restoration with multiple sensors integrated. (ii) Schematic diagram of external data collection and processing.

In contrast, the innovation of this study lies in a synergistic combination of 3D printing technology, a novel biomimetic material system, and smart sensor systems [28–37]. We propose a novel solution that integrates a custom 3D-printed crown fabricated from a P(NiPAAm-bis-AA) hydrogel. The core material innovation is the incorporation of a bioinspired inverse opal structure at the crown–tooth interface (Fig. 1A).

This microporous network, as revealed by scanning electron microscopy (SEM), is designed to significantly enhance the mechanical adhesion and stability between the crown and the tooth by maximizing the contact surface area at the microscopic level. Within this hydrogel matrix, we embed flexible pressure resistance sensors [38–40]. These sensors are constructed with a single-layer graphene-doped polydimethylsiloxane (SLG/PDMS) nanocomposite as the flexible substrate and sensing element, which offers excellent biocompatibility and piezoresistive sensitivity. We hypothesize that integrating this bioinspired inverse opal hydrogel structure with machine learning-enhanced flexible sensors will significantly improve the mechanical coupling accuracy and real-time monitoring capability of dental restorations compared to traditional smooth-interface methods (Fig. 1B).

This integrated system allows the embedded sensors to capture real-time, multidimensional pressure distribution data between the crown and the tooth, overcoming the limitations of existing unidirectional sensing systems. The stability provided by the inverse opal structure ensures that the sensors remain functional despite fluctuations in the oral environment. Furthermore, the collected data are interpreted by machine learning algorithms to enable predictive modeling of occlusal forces. This approach facilitates a paradigm shift toward intelligent, data-driven dental restorations that can be personalized for long-term fit, durability, and patient comfort (Fig. 1C and D).

## Materials and Methods

### Materials

The materials employed in this experiment included epoxy resin, acrylic acid (AAc), a photoinitiator, and a dye. AAc (Sigma-Aldrich) and N-isopropylacrylamide (Sigma-Aldrich) were used to produce the hydrogel employed in this experiment. The crosslinking agent and thermal initiator used *N*,*N*′-methylene bisacrylamide (BIS; Sigma-Aldrich) and 2-hydroxy-2-methyl-1-phenylacetone (HMPP). Hydrofluoric acid solution (HF) is provided by Aladdin Industries. The aqueous solution of reduced graphene oxide (rGO) was provided by Nanjing XFNANO Material Technology Co. Ltd. The surfactant in rGO aqueous solution is polyvinylpyrrolidone. All studies used pure water with a resistivity greater than 18 MΩ cm, filtered through the Milli-Q Plus 185 water purification system (Bedford, MA, USA).

### Printer ink preparation

Epoxy resin, AAc (5 g), dye (1.5 mg), and photoinitiator (75 mg) were added at room temperature. The entire formula is thoroughly mixed in centrifugal mixers (THINKY mixer AR-100 and AR-250) at 400*g* (2,000 rpm) for 5 min.

### Printer

The experiment uses a REMPBD OS printer from Nanjing RuiPu Information Technology Co. Ltd. The molding technology adopts photopolymerization technology, and the light source system adopts a brand new REIIGHT parallel light source system. The upload method uses both USB and Ethernet. The slicing software uses chiubox V1.9.0 and above.

### The specific printing method

First, modeling software such as 3Dmax was used to create a model and then saved in an obj file format; the obj file was imported into the slicing software; the support, printing layer thickness, and other parameters were set; and finally the file was imported into the printer for printing.

### Preparation of P(NiPAAm-bis-AA) inverse opal film

An approach using sacrificial templates was employed to create the P(NiPAAm-bis-AA) inverse opal film. Firstly, NiPAAm (9.72 mmol) and bis (0.81 mmol) were mixed in a molar ratio of 29:1. Then, the mixture was mixed with deionized water (34.7 mmol) to prepare P(NiPAAm-bis-AA) hydrogel solution with a concentration of about 10%. At the concentration of 2 mg/ml, the rGO aqueous solution with different concentrations (1, 2, 3, 4, or 5 mg/ml) is added to the hydrogel solution prepared above. The obtained P(NiPAAm-bis-AA) hydrogel solution was injected into the gap of silica colloidal crystal chips. Before curing, the hydrogel is exposed to ultraviolet light for 100 s through a mask to facilitate the curing process. After curing, the hydrogel outside the membrane is mechanically removed. Finally, hydrofluoric acid (HF) is used to etch the cured hydrogel to produce the required hydrogel film.

### Sensor fabrication

Ti /Au cross electrodes were deposited on an SLG/PDMS substrate using an electron beam evaporator, and contact pads were added at both ends of the electrodes to achieve external circuit connections. Subsequently, the SLG/PDMS surface was treated with oxygen plasma to enhance adhesion. Subsequently, rGO/P(NiPAAm-bis-AA) precursor solution was prepared and stored under refrigeration conditions. The solution was injected between PDMS sheets with staggered electrode design, capillary action was used to make it penetrate into the electrodes, and polymerization and curing were realized through ultraviolet radiation, finally forming composite hydrogel with electrode pattern. This fabrication process results in a composite hydrogel in which the electrode pattern is fully encapsulated, ensuring that the sensing element is isolated from the external environment and potential biological fluids.

## Results and Discussion

**Fig. 2.**
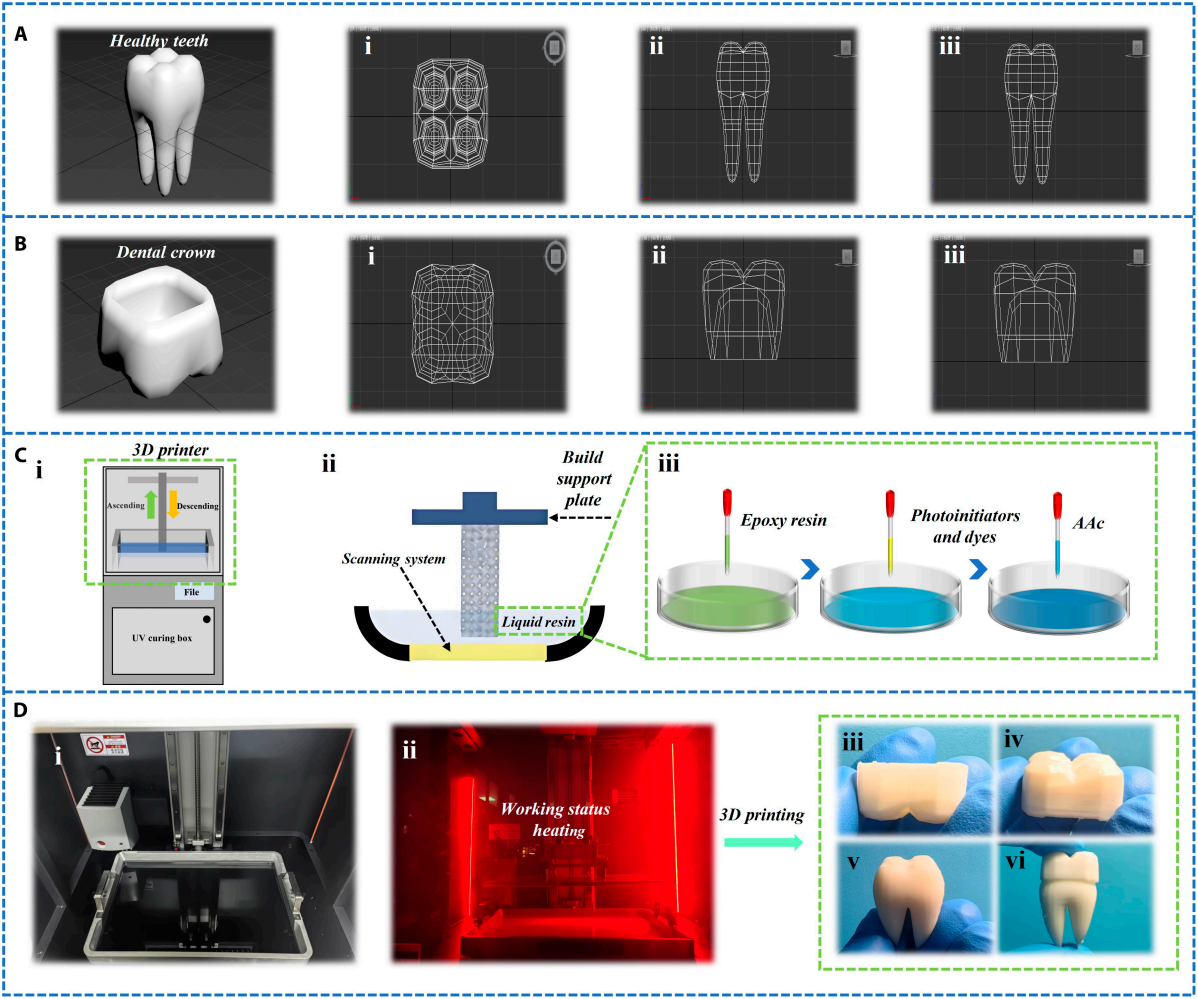
The complete production process of 3D-printed tooth and crown models. (A) Create a 3D digital model of teeth using 3Dmax modeling software, and display its wireframe from a 3-view perspective. Figure shows the top view, front view, and left view of the model, respectively (i to iii). (B) Create a 3D digital model of the dental crown using 3Dmax modeling software, and display its wireframe from a 3-view perspective. Figure shows the top view, front view, and left view of the model, respectively (i to iii). (C) Specific steps of 3D printing. Display of the internal and back structures of the 3D printer, mainly including the printer cover, print head lifting platform, resin tank, curing box, heater, switch, etc. (i). The process of liquid resin depositing layer by layer on the support plate through the print head to form a tooth model (ii). The preparation process of resin materials was demonstrated, including the mixing of epoxy resin, AAc, photoinitiator, and dye (iii). The light curing system inside the printer during the printing process (iv). (D) Status of the printer before and after operation (i and ii). The printed physical images of dental crowns and tooth models (iii to vi).

In the past few decades, the design and manufacturing of dental crowns have evolved from traditional metal materials to more advanced options such as ceramics, resins, and composite materials. These modern materials greatly enhance the aesthetic and functional quality of dental crowns. However, issues related to long-term fit, durability, and comfort still exist. Firstly, this study used 3Dmax software to design tooth models, including dental crowns and tooth models, and presented wireframes of the teeth and crowns from a 3-view perspective (Fig. 2A and B). 3Dmax software is fully functional and flexible in operation, making it easy to customize complex structures of dental crown models and solve complex dental health problems for patients. Then, the 3Dmax file in obj format was imported into the software that comes with the photopolymerization printer for slicing and parameter settings. The highest accuracy of the photopolymerization printer used can reach 0.01 mm per layer, ensuring high-precision customized dental crown models. Finally, the set file was imported into the printer and the solid model was manufactured through 3D printing technology (Fig. 2D). The main structure of a photopolymerization printer includes a print head lifting printing platform, resin tank, curing box, optical scanning system, heater, and switch. The resin used in this study is mainly composed of epoxy resin, acrylic ester, photoinitiator, and dye (Fig. 2C).

**Fig. 3.**
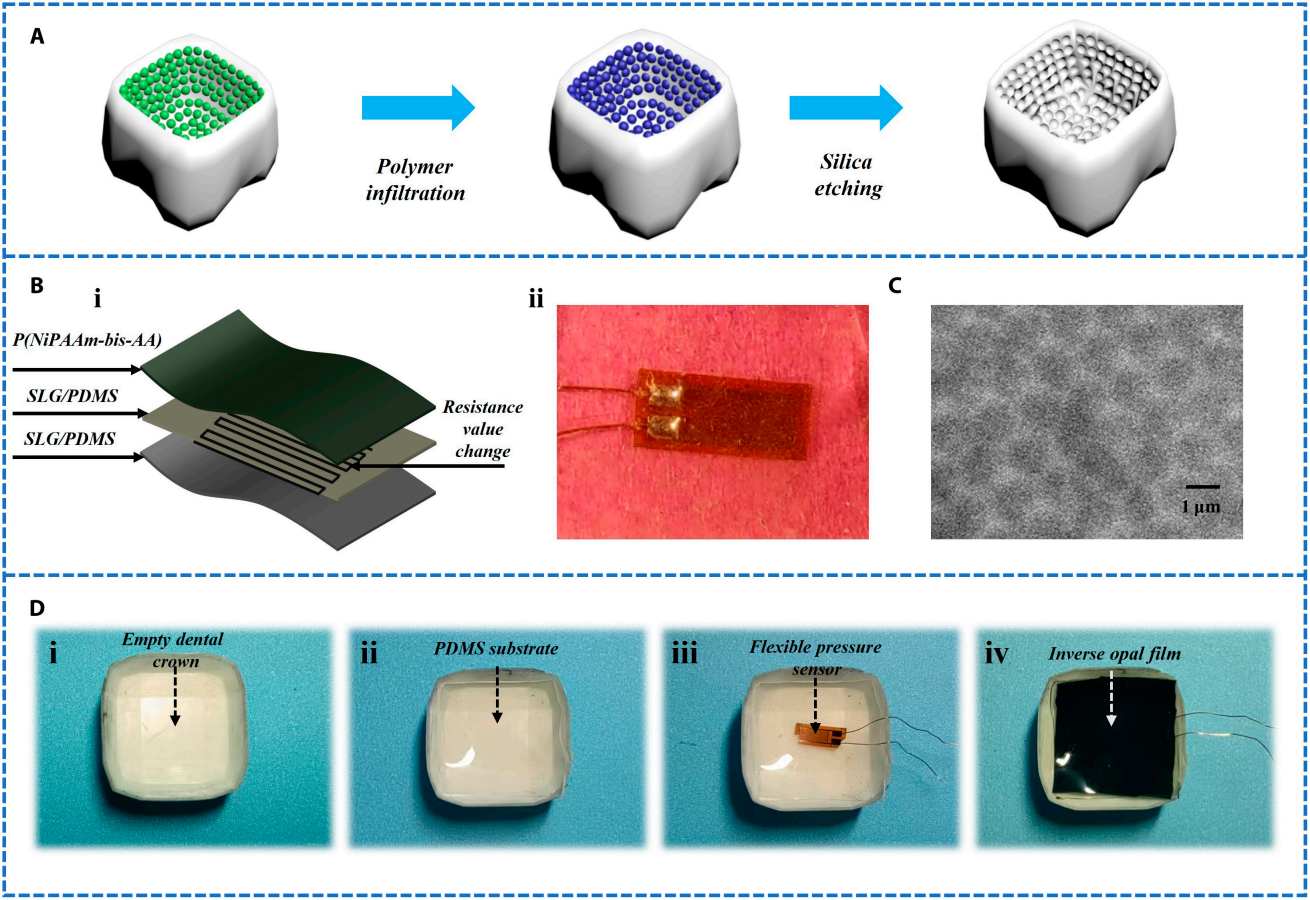
Preparation and sensor integration of the positive and negative surface structures of the tooth model. (A) Preparation process of positive and negative structure treatment on the surface of dental models. (B) Internal structure and physical image of the sensor. (C) SEM image of inverse opal structure. (D) Integration of dental crown models. (i) A basic dental crown model is displayed. (ii) The surface of the model is coated with a layer of PDMS to provide better adhesion. (iii) A sensor was installed on the model, connected to external devices via copper wires, for monitoring or responding to dental conditions. (iv) Encapsulating the model in a layer of inverse opal film, the pore structure of the inverse opal film effectively increases the adhesion of the entire dental crown system.

In order to increase the adhesion between the dental crown and teeth, silica particles were laid on the inner surface of the dental crown model through self-assembly, and then the surface was filled with polymer through polymer infiltration, enhancing the structural integrity of the model. Subsequently, silica etching treatment was performed to form a reverse opal structure, which has a special pore structure that enhances the adhesion between the dental crown and teeth (Fig. 3A).

Flexible sensors are composed of multiple layers of structure, which can detect changes in resistance values caused by pressure. Specifically, the sensor is composed of the SLG/PDMS substrate, a grid-shaped conductive foil (in this study, Kangcu foil was used), a thin SLG/PDMS layer, and a P(NiPAAm-bis-AA) layer (Fig. 3B, i). Among them, PDMS, the main component of the SLG/PDMS substrate, serves as a flexible substrate, improving the biocompatibility of the flexible sensor and enabling it to be better integrated into dental crowns. The use of SLG single-layer graphene enhances the conductivity of the sensor and also increases its mechanical strength, introducing the press-resistive effect. The use of 2 layers of SLG/PDMS enhances the mechanical properties of the entire structure. Among them, the P(NiPAAm-bis-AA) layer endows the sensor with temperature responsiveness, allowing it to undergo volume expansion or contraction when the temperature changes. When the patient’s oral temperature changes, it better adheres to the inside of the dental crown and will not fall off.

The specific preparation process of flexible press-resistive sensors is as follows: In order to prepare the SLG/PDMS substrate, we fully mixed the prepolymer gel (Sylgard 184 silicon elastomer) with the crosslinking agent (Sylgard 184 silicon elastomer curing agent) in the weight ratio of 10:1 and evenly spread it into a thin layer. Then, graphene is dispersed in volatile solvent ethanol and mixed with PDMS prepolymer to evaporate the solvent. Subsequently, the thin layer was placed in a 65 °C oven and heated to cure for 2 h. After curing, the SLG/PDMS substrate was precisely cut into rectangular films with dimensions of 5 mm × 8 mm. To enhance the hydrophilicity of the SLG/PDMS surface, dilute oxygen plasma treatment was used to activate the film surface for 1 min, thereby introducing hydrophilic functional groups. Next, silanization treatment was performed on the SLG/PDMS substrate. The specific steps are as follows: The activated SLG/PDMS film was immersed in a solution containing 3-(trimethoxysilylpropyl) propyl methacrylate (Sigma-Aldrich, product number 440159) for 3 min. After silanization treatment, the SLG/PDMS substrate was thoroughly cleaned with anhydrous ethanol and dried under nitrogen flow. Subsequently, the grid-shaped conductive foil prepared by laser cutting (using copper foil in this experiment) was accurately placed on the processed SLG/PDMS substrate. Two 1 × 1 mm ^2^ solder joints were made at both ends of the conductive foil for connection to external circuits. To protect the conductive foil and improve its stability, a thin layer of SLG/PDMS coating is spin coated on its surface, and then heated and cured again in a 65 °C oven for 2 h. Finally, the surface of the conductive foil covered with SLG/PDMS was treated with dilute oxygen plasma, and then the flexible inverse opal structured photonic crystal film was polymerized onto the surface functionalized conductive foil using the same silanization step as described above. This process ensures good adhesion and excellent performance between the film and the substrate.

**Fig. 4.**
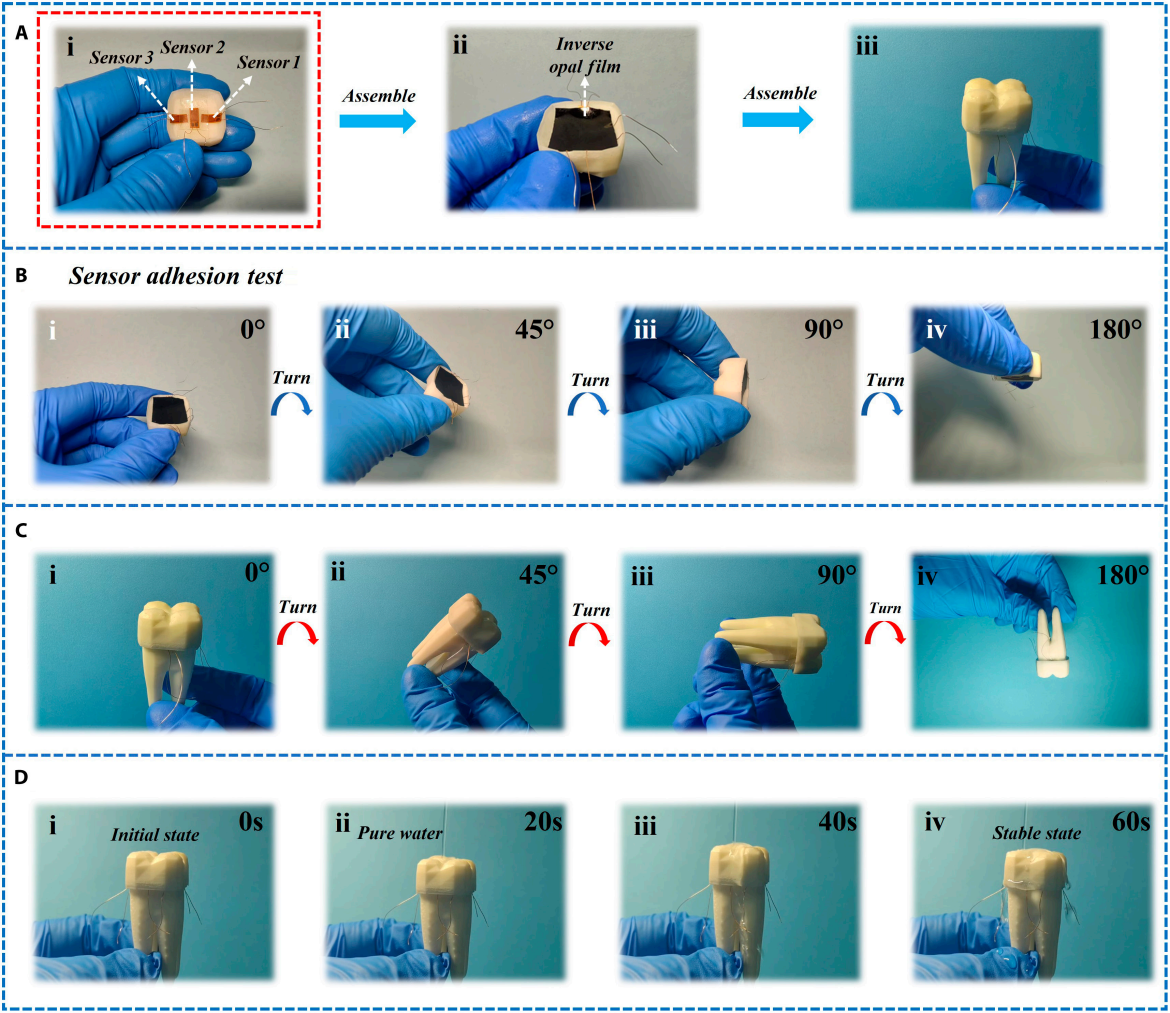
Installation and adhesion test of sensor. (A) The sensor is integrated with the dental crown and installed on the tooth model according to the design concept of triaxial rosette gauge. (i) Install on the tooth model according to the design concept of the three-axis rose ring strain gauge. (ii) Install the anti-protein stone structure film. (iii) Complete the installation on the tooth model. (B) Adhesion testing of dental crown sensors. After combining the crown with the sensor, rotate it from 0° to 45°, 90°, and 180° in sequence (i-iv), observe whether it falls off, and test the adhesion. (C) Adhesion test between dental crown sensor and teeth. After combining the crown sensor with the tooth, rotate it from 0° to 45°, 90°, and 180° in sequence (i-iv), observe whether it falls off, and detect the adhesion force. (D) Adhesion of dental crowns in simulated humid environments. After combining the dental crown sensor with the tooth (i), impact the dental crown with pure water (ii) to simulate the adhesion of the dental crown (iii) in a humid oral environment (iv).

In order to collect real-time and multidimensional data, we have installed sensors at different positions inside the dental crown, which can collect pressure information in 3 directions. The layout of sensors follows the design concept of triaxial rosette gauge and is placed in the main strain directions predicted by finite element analysis: sensor 1 on the buccal side (to withstand the compression of the lateral teeth), sensor 2 on the top of the dental crown (to withstand maximum pressure), and sensor 3 on the outer side of the teeth (to withstand shear stress) (Fig. 4A, i). In order to increase the adhesion between the dental crown and teeth, we applied a layer of inverse opal structure film on the surface where the final dental crown contacts the teeth (Fig. 4A, ii). Finally, the processed dental crown will be placed on the tooth model to form the final dental restoration and pressure detection system (Fig. 4A, iii).

In order to verify the good adhesion of the entire system, 3 sets of adhesion tests were conducted separately. Firstly, the rotation test of the dental crown model at different angles (0°, 45°, 90°, and 180°) is conducted to verify the stability of the sensor on the dental crown. Each image shows the state of the sensor during the rotation of the tooth model, and even after 180° rotation, the sensor remains firmly adhered to the tooth model without falling off (Fig. 4B). After attaching the dental crown sensor to the tooth model, rotation tests were conducted at different angles (0°, 45°, 90°, 180°) to verify the stability between the dental crown sensor and the teeth. Throughout the entire rotation process, the dental crown sensor remained on the tooth model without detachment, demonstrating its good adhesion (Fig. 4C). The state of the tooth model at different time points (20, 40, 60, and 80 s) under water impact was recorded starting from 0 s. Throughout the entire testing process, the sensors on the tooth model remained firm and showed no signs of detachment, demonstrating its stability and adhesion under humid conditions (Fig. 4D).

Our adhesion testing strategy is specifically designed to simulate the complex multi-axis mechanical challenges encountered in clinical oral environments, which is significantly different from the controlled unidirectional forces measured by standard lap shear or peel tests. The main challenge in applying these standard tests directly to our system is the complex nonplanar geometry of the dental crown itself. Overlapping shear and peel testing require flat, parallel bonding surfaces to generate meaningful and comparable data. However, the complex inner surface of dental crowns is designed to precisely adapt to the irregular terrain of prepared teeth, making it exceptionally difficult to prepare standardized samples for such tests without fundamentally altering the functional geometry of the crown and the integrity of the bonding interface (Fig. S1).

To enhance the rigor of adhesion assessment, we extended the adhesion evaluation to physiological relevant aqueous substitutes for testing. Specifically, after immersing the materials in artificial saliva at 37 °C for 180 d, we retested their adhesion (Fig. S2). We adopted rotation and high-pressure water impact tests as more clinically relevant and functional evaluations. These methods directly simulate key real-world scenarios: The rotation test evaluates the stability of the dental crown interface under torsional and off-axis forces, indicating challenges in food handling or quasi-functional habits. The high-pressure water impact test evaluated the adhesive strength under high shear stress conditions mimicking swallowing or drinking water, as well as humid oral environments (Fig. S3).

**Fig. 5.**
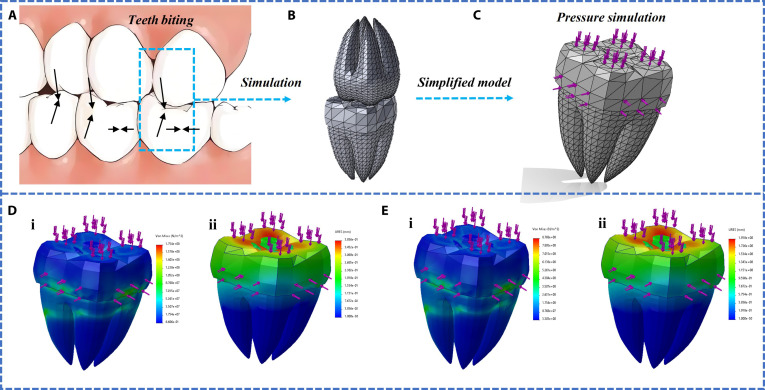
Mechanical simulation of teeth and dental crowns. (A) Dental bite diagram. (B) Independently create a 3D model of 2 teeth biting up and down, with one tooth equipped with a crown sensor. (C) Mesh division and simulation force diagram of tooth model. (D) Simulation results of tooth force under 100-N pressure. (i) Stress distribution of teeth under stress. (ii) Displacement distribution of teeth under stress. (E) Simulation results of tooth force under 500-N pressure. (i) Stress distribution of teeth under stress. (ii) Displacement distribution of teeth under stress.

In order to accurately simulate the mechanical behavior of dental crowns during occlusion, we conducted static structural finite element analysis. The key parameters used in the simulation are as follows: The tooth model is manufactured through photopolymerization 3D printing, and the resin material used has been tested by a universal testing machine. Its Young’s modulus is about 2.5 GPa, and the Poisson’s ratio is set to 0.3. P(NiPAAm-bis-AA) hydrogel used in this study has remarkable flexibility. According to the tensile test results, due to the almost incompressibility of the material, the Young’s modulus is set to 5 MPa and the Poisson’s ratio is set to 0.49. It is assumed that there is complete adhesion between the hydrogel and the resin. As shown in Fig. 5C, in order to simulate the fixed state of teeth in the alveolar bone, the root base of the tooth model is set as a fixed constraint. This is the standard boundary condition setting method in dental biomechanical simulation. The load application method refers to the common direction of clinical biting force. A vertical downward load of 100 or 500 N was applied evenly to the occlusal surface of the dental crown (indicated by the pink arrow) to simulate the static pressure during the occlusion process of upper and lower teeth. The main output of this simulation is the directional strain component, especially the strain in the *X*, *Y*, *Z* Cartesian coordinate system. This directly corresponds to the layout of the 3 directional sensors (sensors 1 to 3) in our experiment, making it easy to directly compare the simulation results with the resistance change signals of the sensors. This analysis did not directly use the principal strain, as the directional component more intuitively reflects the deformation in a specific direction of the sensor.

The force analysis of 2 teeth was selected when biting up and down, and the force was analyzed in 3 directions when teeth bite. Taking the following teeth as an example, they represent the compression of the buccal side by the lateral teeth during biting, the maximum pressure on the crown top, and the shear stress on the outer side of the teeth (Fig. 5A to C). According to the literature review, the average pressure during tooth occlusion is 100 to 500 N. Therefore, we simulated the dental crown model using pressures of 100 and 500 N (Fig. 5D and E). According to the simulation results, when the pressure is the same, the displacement at the top of the dental crown is the largest, followed by the side, and the side is the smallest. As the pressure increases, the displacement of all positions relatively increases.

**Fig. 6.**
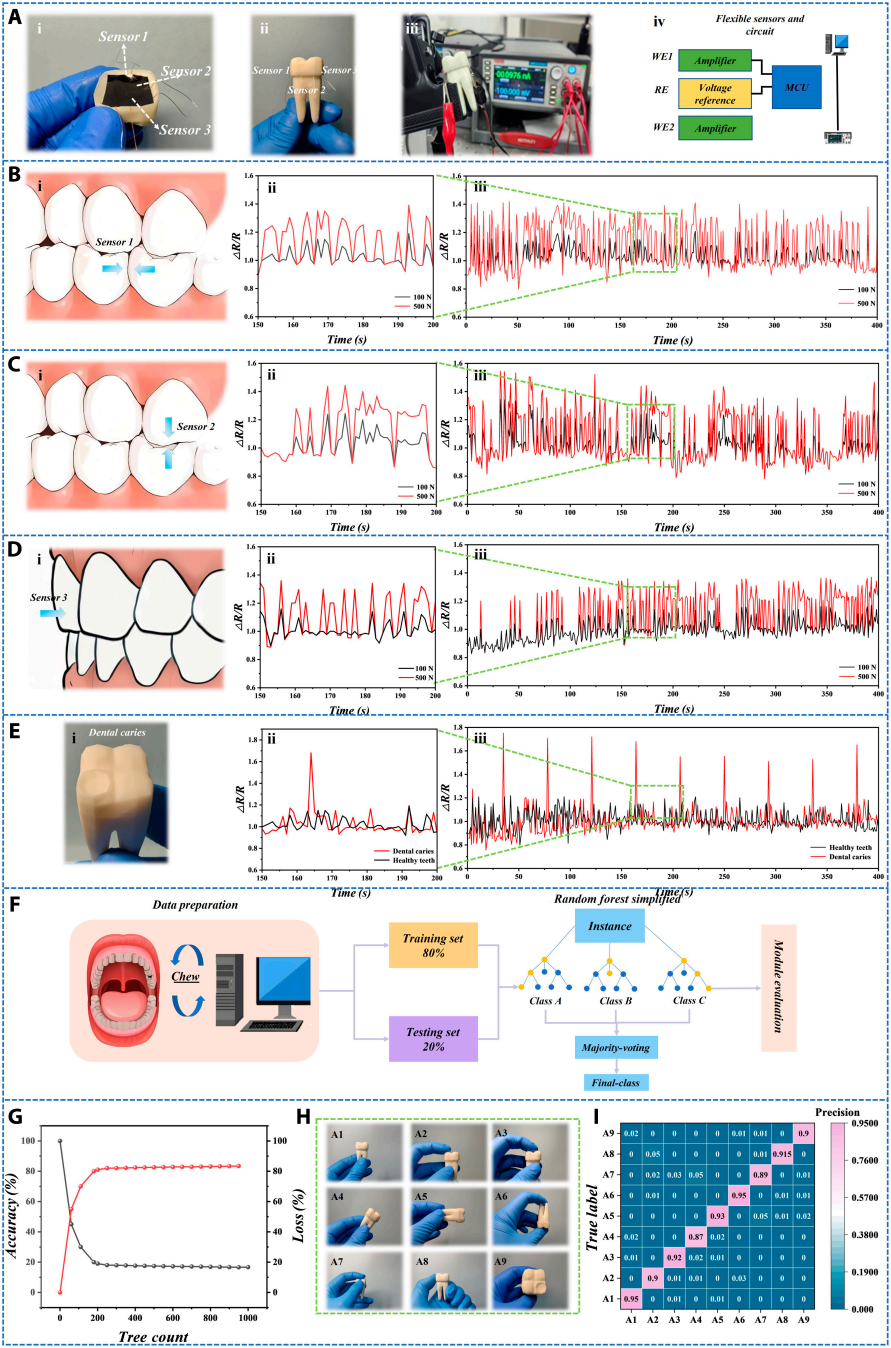
Testing and data analysis of dental sensors. (A) Differential labeling of internal sensors in dental crowns, experimental circuit connections, and schematic diagram of external data collection and processing. (i, ii) Differential labeling of internal sensors in dental crowns. (iii) The experimental circuit connection. (iv) Schematic diagram of external data acquisition and processing.(B to D) Under different pressure conditions, the fluctuation curves of the relative resistance values measured by the sensors at 3 positions over time reflect the response of the sensors to the stress on the teeth. (B) (i) Schematic diagram of the sensor being stressed at the buccal side of the crown. (ii, iii) Fluctuation curves of relative resistance values over time when applying 100N and 500N pressure to the sensor at the buccal side. Note that (ii) is a partial magnification of (iii). (C) (i) Schematic diagram of the sensor being stressed at the top of the crown. (ii, iii) Fluctuation curves of relative resistance values over time when applying 100N and 500N pressure to the sensor at the top of the crown. Note that (ii) is a partial magnification of (iii). (D) (i) Schematic diagram of the sensor being stressed at the outer side of the crown. (ii, iii) Fluctuation curves of relative resistance values over time when applying 100N and 500N pressure to the sensor on the outer side of the crown. Note that (ii) is a partial enlargement of (iii). (E) In the case of dental caries, the fluctuation curve of relative resistance value over time was measured. (i) Physical image of the caries model, showing caries occurring on the top of the tooth. (ii, iii) Fluctuation curves of the relative resistance values over time for healthy teeth and caries teeth, under the same pressure applied to the sensor at the same location. (F) The process of random forest algorithm mainly includes data preparation, model training and testing, and model evaluation. (G) Relationship between the number of trees and accuracy. (H) A1 to A9 represent different health states of tooth occlusion or different positions under the same health state. (I) Confusion matrix diagram is used to evaluate the performance of classification models.

As shown in the force analysis of the dental crown (Fig. 5C), the 3 areas where the teeth are subjected to force are represented by sensors (Fig. 6A), with sensor 1 on the buccal side (subjected to compression from the lateral teeth), sensor 2 on the top of the dental crown (subjected to maximum pressure), and sensor 3 on the outer side of the teeth (subjected to shear stress). This corresponds one-to-one with Fig. 4A (i). Then, the sensor was connected to an external device and the resistance changes were recorded at 3 different positions [Fig. 6B (i), C (i), and D (i)].

The principle of flexible pressure resistance sensors is based on the pressure resistance effect. When the sensor is subjected to external force, the conductive material inside will deform, causing a change in resistance value. Specifically, flexible pressure-resistant sensors are typically composed of elastic polymers and conductive materials. Elastic polymers provide deformability, while conductive materials affect the electrical performance of sensors. When the sensor is subjected to external stress, the length and cross-sectional area of the conductive material will change, resulting in a change in resistance, which can be detected by the circuit. In order to specifically test the response of the flexible pressure-resistant sensor under different external forces, the dynamic response of the relative resistance value (Δ*R*/*R*) of the flexible sensor over time was measured under different pressure conditions (100 and 500 N). At the same position, as the pressure increases, the relative resistance value will also increase. At the same pressure level, the relative resistance value of sensor 2 at the top of the dental crown has the largest variation range, followed by sensor 1 on the buccal side, and sensor 3 on the outer side of the dental crown has the smallest variation range (Fig. 6B to D). This is consistent with the conclusion we reached during the simulation process. When dental caries occur, the relative resistance value measured by the sensor always undergoes sudden and excessive changes (Fig. 6E). Finally, we verified the long-term stability of the sensor to ensure the accuracy of experimental data (Fig. S4).

The tooth bite signals measured above were identified and predicted for state and position using machine learning methods. A random forest model was used to calculate the average and standard deviation of the relative resistance values measured for each signal, which were used as input features for the random forest model. Random forest is one of the representative methods of ensemble learning, which constructs multiple decision trees and classifies or averages them to obtain the final results. It has the advantages of anti-overfitting, high accuracy, robustness, parallelizability, interpretability, etc. The diversity of multiple trees reduces the bias of a single tree and is suitable for nonlinear data. It has a certain tolerance for missing values, outliers, and noisy data and can be independently trained between trees, making it suitable for distributed computing. The task flow of the random forest algorithm mainly consists of 3 steps: data preparation, model training and testing, and model evaluation (Fig. 6F). By using data partitioning (80% training, 20% testing) and ensemble learning methods with multiple decision trees, the high accuracy of the model is ensured. This model has a wide range of application scenarios, including credit scoring, disease monitoring and diagnosis, and predicting weather and ecosystem changes. The accuracy of the random forest algorithm rapidly increases with the increase of decision trees, reaching a stable value around 200 decision trees, and then slowly increases (Fig. 6G). A1 to A9 represent different tooth states (Fig. 6H). The random forest model performs well in classifying bite signals of 9 teeth in different states or at different positions of the same state, with an accuracy rate of 0.87 or above. The confusion matrix is an important tool for evaluating model performance in classification tasks, which intuitively displays the correspondence between model prediction results and real labels (Fig. 6I).

## Conclusion

This study presents an innovative approach integrating 3D-printed hydrogel dental crowns with embedded flexible pressure sensors for real-time occlusal force monitoring. By employing an inverse opal structure, we significantly enhance the adhesion and stability of the hydrogel crown, ensuring long-term durability in the oral environment. The embedded pressure resistance sensors effectively capture multidimensional occlusal force distribution, providing valuable real-time data for personalized dental restorations. Furthermore, a machine learning-based classification model, utilizing a random forest algorithm, accurately predicts occlusion states with an accuracy exceeding 87%. The integration of advanced biomaterials, smart sensing technology, and artificial intelligence demonstrates a promising direction for intelligent dental healthcare, paving the way for more precise, reliable, and patient-specific dental restoration solutions. Future research will focus on optimizing sensor sensitivity, improving biocompatibility, and expanding clinical applications to further enhance real-time oral health monitoring systems.

It is worth noting that P(NiPAAm-bis-AA) hydrogel and PDMS materials used in this system have shown good biocompatibility and low inflammation in existing studies. For example, research shows that PNiPAAm-based hydrogel can effectively reduce the aggregation of macrophages and the secretion of proinflammatory cytokines [such as tumor necrosis factor-α (TNF-α)] in the subcutaneous implantation model [5]. Similarly, PDMS, as a highly biologically inert material, has a low risk of causing severe foreign body reactions or chronic inflammation in long-term medical implant applications [6]. These literature evidences provide preliminary and positive theoretical basis for the biosafety of our prototype.

## Data Availability

The authors confirm that the data supporting the findings of this study are available within the article and its supplementary materials.
