# Supplementary material for: 3D-Printed Colloidal Crystal Hydrogel Crown Fused with Machine Learning-Integrated Resistance Strain Sensor for Pressure Sensing
Source: Biomater Res. 2026 Feb 4;30:0313. doi: 10.34133/bmr.0313 (PMC12868556; doi:10.34133/bmr.0313)
Supplement: Supplementary 1 — Figs. S1 to S4 [file bmr.0313.f1.docx]

**Supporting information**

**3D printed colloidal crystal hydrogel crown fused with machine learning integrated resistance strain sensor for pressure sensing**

*Zheng Mao,^a^*^†^ *Dongxiang Yang, ^b^*^†^ *Ling Tang, ^b^*^†^ *Qing He,^a^ Yue Wang, ^a^ Songchao Fu, ^a^ Zhiwei Jiang,^a^ Ying Wang,^a^ Chenkai Zou,^a^ Cihui Liu,^a^*, Linling Yin ^c^**

a Center for Future Optoelectronic Functional Materials, School of Computer and Electronic Information/School of Artificial Intelligence, Nanjing Normal University, Nanjing, 210046, China.

b Department of Nursing, Shanghai General Hospital, Shanghai Jiao Tong University School of Medicine, Shanghai, 201600, China.

c Department of Stomatology, Shanghai General Hospital, Shanghai Jiao Tong University School of Medicine, Shanghai, 200080, China

† These authors contributed equally to this work.

*Corresponding Authors:

Cihui Liu: cihui@njnu.edu.cn; Linling Yin: yinlinling12130@163.com


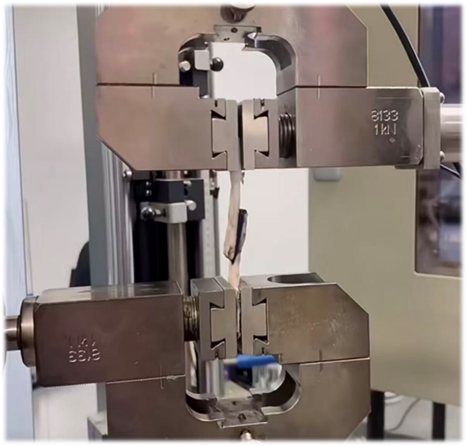


**Figure S1:** Lap‑shear test.


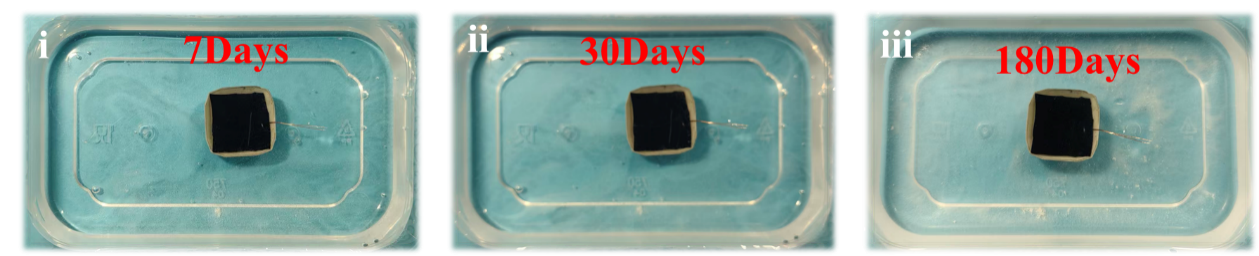


**Figure S2.** Long term immersion of dental crowns in artificial saliva.


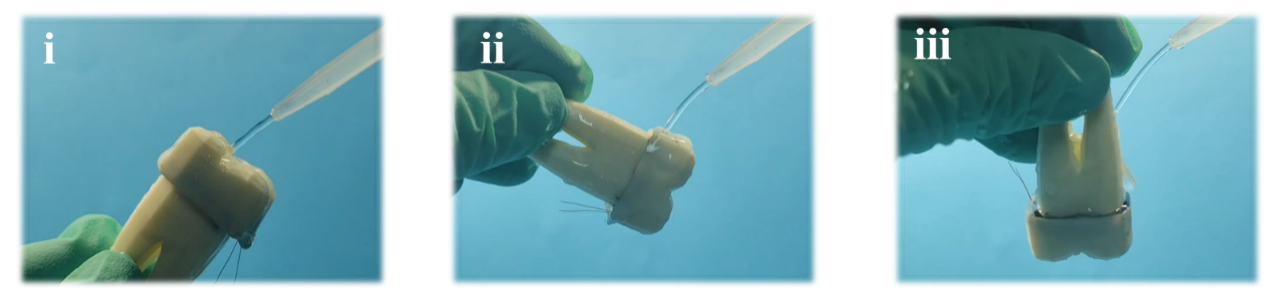


**Figure S3.** After soaking the dental crown in artificial saliva for a long time, remove it and perform adhesion testing again.


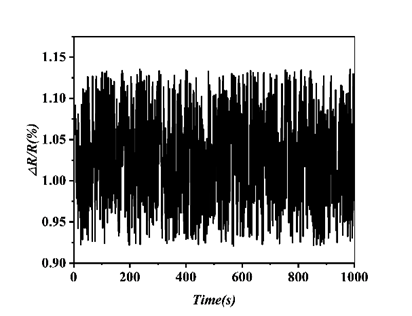


**Figure S4.** Stability measurement of sensors after long-term artificial saliva immersion.
